# Status Epilepticus Mortality Risk Factors and a Correlation Survey with the Newly Modified STESS

**DOI:** 10.3390/healthcare9111570

**Published:** 2021-11-18

**Authors:** Tzu-Hsin Huang, Ming-Chi Lai, Yu-Shiue Chen, Chin-Wei Huang

**Affiliations:** 1 Department of Neurology, National Cheng Kung University Hospital, College of Medicine, National Cheng Kung University, Tainan 70101, Taiwan; oxlesson@gmail.com (T.-H.H.); snow700709@gmail.com (Y.-S.C.); 2 Department of Pediatrics, Chi-Mei Medical Center, Tainan 70101, Taiwan; vickylai621@gmail.com

**Keywords:** antiepileptic drugs, status epilepticus, STESS, risk factors, score

## Abstract

Background: Status epilepticus (SE) is a neurological emergency and is usually associated with significant morbidity and mortality rates. Several clinical scales have been proposed to predict the clinical outcome of such incidents, including the Status Epilepticus Severity Score (STESS), the modified STESS (mSTESS), and the Encephalitis-Nonconvulsive Status Epilepticus-Diazepam Resistance-Image Abnormalities-Tracheal intubation (END-IT). Nevertheless, there is still a need for a more practical and precise predictive scale. Methods: This is a retrospective cohort study which examines data from patients with SE in our Department of Neurology between 2009 and 2020. Based on the outcome of each case, the patients were divided into survivor and non-survivor groups. We analyzed the independent factors and adjusted the STESS to achieve a better prediction of prognosis. The predictive accuracy of our new STESS scale was then compared with that of the mSTESS and the END-IT. Results: Data on a total of 59 patients were collected, with 6 of them classified as non-survivors. The effects of the variables of age, sex, underlying disease(s), and type(s) of antiepileptic drug (AED) use showed no significant differences between the survivor and non-survivor groups. Importantly, the number of AEDs used in the first week and the use of thiobarbiturates predicted non-survival. We adjusted the STESS to create the newly modified STESS (nSTESS), which showed a better predictive capacity than the STESS, the mSTESS, and the END-IT. Conclusions: Our adjustment of the STESS with the addition of the factors “number of AEDs within the first week” and “use of thiobarbiturates”, could have a positive impact on the prediction of mortality rates compared with currently used scales. This nSTESS could potentially be useful in clinical practices, for the early prediction of outcomes for patients with SE.

## 1. Introduction

Status epilepticus (SE) is a neurological emergency with relatively high mortality and morbidity rates. The mortality rate for adults who present with a first episode of generalized convulsive status epilepticus (GCSE) is as high as 16 to 20 percent [1]. Numerous factors have been shown to determine the outcome of SE [2], and although etiology is the most important predictor of outcome, old age and medical comorbidity are also independent risk factors for mortality [3].

Currently, there are several published scales for predicting the prognosis of SE: the Status Epilepticus Severity Score (STESS) [2,4]; the modified STESS (mSTESS) [5]; the Epidemiology-based Mortality score in Status Epilepticus (EMSE) [6], and the END-IT score (Encephalitis, Nonconvulsive Status Epilepticus (NCSE), Diazepam Resistance, Image Abnormalities, Tracheal intubation) [7]. Each of these tools have specific advantages and limitations [8].

The STESS has undergone external validation, and remains the most straight forward of these prognostic scales, as it can be used on admission to guide early treatment. Nevertheless, the effectiveness of the STESS is limited by a ceiling effect for patients older than 65 years old. Other characteristics of the STESS that have been demonstrated in previous studies are fair sensitivity (76%) and specificity (62%), poor positive predictive value (PPV) (22–39%), and good negative predictive value (NPV) (81–100%), regarding in-hospital deaths [8].

The other scales partially compensate for the shortcomings of the STESS, but have limitations of their own. The mSTESS, for example, decreases the ceiling effect of the STESS for patients older than 65 years of age, but does not take into consideration the etiologies of SE, and has not undergone external validation. As for the EMSE, it may appear to be superior to the STESS, but the calculations involved are relatively complex to carry out in clinical circumstances, and it does not evaluate NCSE [9]. Finally, the END-IT has been shown to be more accurate in predicting 3-month morbidity, but cannot be used on admission, and has not undergone external validation [8]. Therefore, there is still a need for a more precise and practical predictive scale.

In this study, we identified the major factors involved in making a prognosis in the early stages of SE, and evaluated the predictive value of the STESS. We then aimed to adjust the STESS with the help of the major predictive factors we singled out, in order to improve its predictive accuracy.

## 2. Materials and Methods

### 2.1. Patients

This was a retrospective cohort study. We investigated data on patients with SE from the database at the Neurology Department of the National Cheng Kung University (NCKU) Hospital. The admission dates fell between September 2009 and March 2020. Data on a total of 59 adult patients diagnosed with either convulsive or nonconvulsive status epilepticus were collected. As we work at a medical center, we followed our own protocols and the Taiwan Epilepsy Guidelines to treat patients with SE in the emergency department, the intensive care unit, and the ordinary wards in the Department of Neurology. All patients were diagnosed, treated, and discharged from either the neurology ward or the intensive care unit.

The protocol for treating SE in the NCKU Neurology Department stipulates the initial use of diazepam or midazolam, followed by the addition of one or more standard antiepileptic drugs (AEDs), depending on the semiology, the renal/hepatic function, and the existence of patient comorbidity. In the event of persistent seizures over the following minutes to hours, the protocol stipulates that uninterrupted EEG monitoring should be done as sedative agents such as midazolam, propofol, and thiobarbiturates are continuously administered, in order to achieve a burst-suppression pattern or the total suppression of seizure activity.

### 2.2. Data Collection

SE was operationally defined as “≥5 min of continuous seizure or two or more discrete seizures between which there is incomplete recovery of consciousness” [10]. In addition, based on the definition proposed in 2015 by the International League Against Epilepsy (ILAE)—“the time point of operational dimension 1 (t1) determines the time at which treatment should be considered or started, whereas the time point of operational dimension 2 (t2) determines how aggressively treatment should be implemented to prevent long-term consequences”—5 mins and 30 mins were chosen as the time points t1 and t2, respectively [11]. We categorized the data from patients admitted to our hospital during the 11-year period under observation, based on the diagnosis of SE made following the 1999 or the 2015 ILAE criteria.

The type of SE (convulsive or nonconvulsive), the seizure semiology, the level of consciousness before treatment, and the previous history of seizures/epilepsy were documented upon the patient’s arrival at the department of emergency by interviewing the patient’s family and reviewing the patient’s medical history.

The type of seizure experienced by each patient was identified according to documented clinical data prior to treatment and categorized as a focal aware seizure, a focal impaired awareness seizure, a focal to bilateral tonic-clonic seizure, a generalized seizure, or an NCSE, which had been diagnosed by means of both the clinical diagnosis of a neurologist and EEG data. The level of consciousness of the patient prior to treatment was categorized as alert, somnolent, stuporous, or comatose. The clinical outcome of each case was assessed at hospital discharge.

The STESS is designed to evaluate various patient characteristics. In its recording of age, patients under 65 years of age are assigned a value of 0, and a value of 2 is given to those 65 or older. Patients who have a previous history of seizures are given a value of 0, whereas those without such a history are scored as 1. The severity of patients’ seizures is scored based on the seizure type (focal aware, focal impaired awareness, or focal to tonic-clonic generalized = 0; generalized convulsion = 1; NCSE in coma = 2). Finally, the patients’ level of consciousness is scored as follows: a value of 0 for alert or somnolent, and a score of 1 for stuporous or comatose. An overall score of 0–2 is defined as favorable, indicating a lower risk of death, and a score of 3 points or more is defined as unfavorable and considered a sign of a high likelihood of death.

Information on the following parameters was carefully collected: age; sex; underlying disease(s); admission date; duration of admission; type(s) of SE; type(s) of AED used initially during the first week; time of seizure onset and end; duration of seizure; EEG signal classification of seizure; STESS score, and level of consciousness on admission. In addition, prognosis data was determined on the basis of the clinical outcome (survival or non-survival) at discharge. The patients were divided into survivor and non-survivor groups, which were then compared on all of the above parameters to identify, specifically, which ones showed significant differences between the groups.

### 2.3. Statistical Analysis

Statistical analyses were carried out with SPSS Statistics for Windows, version 20.0 (IBM, Armonk, NY, USA). We used the Pearson’s chi-squared or the Fisher’s exact test to assess the effect of the following categorical variables: sex; underlying diseases (i.e., a history of intracranial hemorrhage, of meningoencephalitis, or epilepsy); seizure type, and treatment (the individual AED or sedative used). We also used the Student’s t-test and the Mann–Whitney U test for the numerical variables (age, number of AEDs used, and STESS score).

The effects of the independent factors were then assessed by conducting a binary logistic regression analysis on the factors associated with higher mortality. Receiver-operating characteristic (ROC) curves were created to assess the ability of these factors, and the ability of the STESS and the newly modified STESS (nSTESS) to predict death. The optimal cutoff point was determined on the basis of the Youden Index and the ROC curve, resulting in adjusted factors that were then added to the STESS to produce the nSTESS. The area under the ROC curve was used to estimate the predictive capacity. The Hosmer–Lemeshow test was used to assess the goodness of fit.

## 3. Results

The comparison of the general characteristics of the survivors and non-survivors is shown in Table 1. We examined data on 59 patients, whose ages ranged from 18 to 93 years old (mean = 55.20, SD = 20.95). Female patients made up 39% of the sample. The mean age was 55.04 (SD = 21.327) for the survivor group and 56.67 (SD = 18.981) for the non-survivor group. There was no significant difference between these two groups in terms of age and sex.

No significant effect was detected for the underlying diseases of meningoencephalitis (*p* = 0.583) or intracranial hemorrhages (*p* = 0.357). The seizure types were similar in the two groups (*p* = 0.115), as was the number (in percentage terms) of patients who were given the following AEDs: valproic acid (*p* = 0.696), levetiracetam (*p* = 0.157), phenytoin (*p* = 0.696), topiramate (*p* = 0.114), lacosamide (*p* = 0.418), and perampanel (*p* = 0.313). Regarding the use of sedative drugs, our data showed no differences for midazolam (*p* = 0.53) or propofol (*p* = 0.108), whereas significant differences were present for thiobarbiturates (*p* = 0.009). There were also significant differences in the number of AEDs used in the first week (*p* = 0.016). The STESS scale was applied to the data but showed no significant differences (*p* = 0.117) between the survivors and the non-survivors.

The multivariate statistical analysis showed that the number of AEDs administered in the first week and the use of thiobarbiturates had a significant impact on mortality. On the other hand, the binary logistic regression analysis examining the relationship between the number of AEDs used and the STESS score (the independent variables) and the outcome of mortality (the dependent variable) showed a significant association between the number of AEDs used and the outcome of mortality (*p* = 0.031), but not in the case of STESS (*p* = 0.143). The beta coefficient of the former was 2.797, and that of the latter was 1.851.

**Figure 1:**
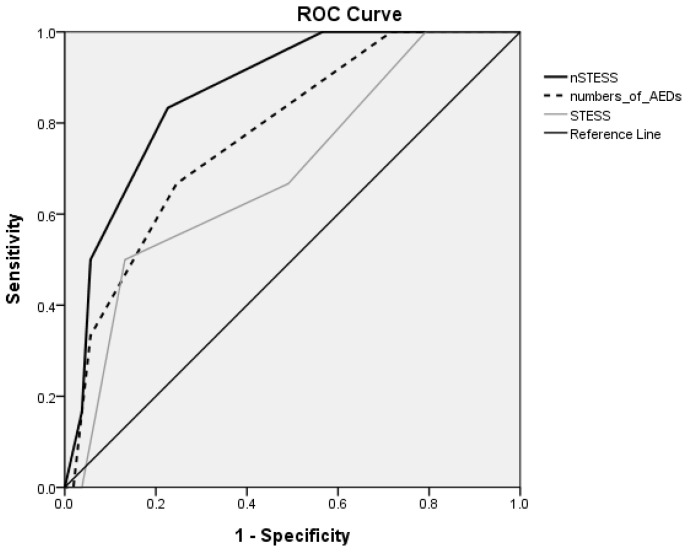
ROC curves of nSTESS, numbers of AEDs, and STESS in our patients. AED, antiepileptic drug; ROC, receiver operating characteristic; STESS, Status Epilepticus Severity Score; nSTESS, newly modified STESS.

The area under the curve (AUC) for the number of AEDs used was calculated as 0.777, with a significant effect (*p* = 0.027) on mortality. The cutoff point was estimated to be located at 2.5 AEDs used, with a maximum value of the Youden Index of 0.421. We assigned a score of 0 when 2 or fewer AEDs were used, and a score of 1 when 3 or more AEDs were used. If thiobarbiturates were used, the score was 1, and it was 0 if they were not used. The point was then added into the STESS to comprise the nSTESS (Table 2). The ROC curve of the nSTESS had a *p*-value of 0.003 and a predictive capacity of 0.868. On the other hand, the ROC curve of the mSTESS in our study had a *p*-value of 0.126 and predictive capacity of 0.692 (Figure 1). Figure 1 shows that the predictive power of the STESS score is similar to that of the use of 2 AEDs in the first week, and that their predictive capacity was even better when they were combined, with an overall accuracy of 86.8%. We selected the cutoff points at the maximum value of the Youden index of 4 for the mSTESS, and 3 for the END-IT. They were very close to the reference line in Figure 1 when at their best predictive cutoff point, and thus we deleted the curves representing these two scales to make the contrast clearer between the STESS (the original) and the nSTESS.

The values of the sensitivity, specificity and predictive capacity of the nSTESS, STESS, mSTESS, and END-IT scale are shown in Table 3. There was a significantly better sensitivity and specificity of nSTESS with a cutoff point of 4 (sensitivity: 83.3%, specificity: 77.4%) compared with mSTESS (sensitivity = 50%, specificity = 56.6%) and the END-IT (sensitivity = 50%, specificity = 66%).

## 4. Discussion

In this study of data from patients with SE who came to the Neurology Department of the National Cheng Kung University Hospital in Tainan, Taiwan, between 2009 and 2020, we did not find a significant difference between survivors and non-survivors for the following variables: age; sex; previous history of intracranial hemorrhage or meningoencephalitis; type of AED, or sedative agents used. However, a significant difference was found between these groups with regards to the number of AEDs used in the first week and the use of thiobarbiturates.

Age is considered a significant factor in accurately predicting patient outcomes on the basis of the STESS [2]; however, it did not have a significant effect in our study. The non-survivor group in our study showed a bimodal age distribution, with one peak at around 33–45 years of age and the other one at around 65–80 years of age. We discovered that the younger group included two cases of new-onset refractory status epilepticus (NORSE). This suggests the possibility that the etiologies in the non-survivor group differed from those in the survivor group. Therefore, it is possible that the presence of the NORSE cases and the relatively small sample size accounted for the absence of a significant effect of age in the STESS in our study.

No patients in the non-survivor group were administered either 1 or 5 AEDs during the first week, and the same number of patients received 2, 3, and 4 AEDs at this time; thus, the distribution of the number of AEDs administered to the non-survivor group differed significantly from that of the survivor group. In the case of the latter, the largest number of people received 1–3 AEDs during the first week, with a cutoff value of 3.5 on the ROC curve. Related to this point is the possibility that, in cases of severe SE, the clinician might shift to the intravenous administration of sedative agents at an earlier stage, instead of continually adding AEDs during the first week.

There is evidence in animal models that applying early combination therapy to the treatment of SE provides better seizure control than using single therapy [12,13]. In studies on animal models conducted in 2019 and 2020, it was shown that the early simultaneous administration of a midazolam–ketamine–valproate combination effectively suppressed SE [14,15]. However, to date, there have been no definite statements regarding the use of early polytherapy in clinical guidelines, and the existing clinical research on early polytherapy for convulsive SE is limited [16]. Nevertheless, the finding in our study that the more AEDs used in the first week, the worse the outcome, suggests that early polytherapy may only benefit SE which is easier to control, than both refractory SE and super-refractory SE. This finding further indicates that the administration of a high number of AEDs is a sign of the presence of severe seizures or SE, which could be associated with the clinical outcome. These results show that additional prospective clinical studies on the use of early combination therapy in the treatment of SE are warranted.

Barbiturates were first synthesized in 1864, and have been used to stop acute seizures since the 1900s [17]. Among these drugs are thiobarbiturates, an anesthesia that is effective in treating SE; however, these commonly cause respiratory depression and myocardial hypotension. In our protocol, thiobarbiturates were used as a third-line anesthetic agent after the propofol treatment had failed, as a result either of treatment failure or of complications. The use of thiobarbiturates was associated with a higher mortality rate, and therefore they were added to the nSTESS, with a value of 1 assigned to cases in which thiobarbiturates were used. This is consistent with the notion that the eventual use of a third-line anesthetic agent such as thiobarbiturates indicates a higher probability of mortality.

We observed that the primary factor behind the mortality in the non-survivor group was the failure of the propofol treatment, and the use of thiobarbiturates in these cases did not usually provide a better therapeutic effect. Therefore, the application of this new scale in countries or areas where protocols call for thiobarbiturates to be used earlier than propofol should be done with caution.

The comparison of the mortality rate observed in our study to that of other studies showed a relatively better outcome in our hospital (10.1%) than the global range of 14.9–20% [18]; this shows our treatment protocol to be effective in dealing with SE and NCSE. The nSTESS we have designed highlights the importance of using a scale that is straight forward in order to predict the outcome of SE. In addition to the good predictive capacity observed so far, our scale is a dynamic scale which takes into account the components of various underlying conditions of the patient, and the severity of the seizure in relation to the patient’s response to second-line AEDs. Nevertheless, owing to the small size of the non-survivor group in our study, the randomization into different datasets for further validation would have a significant impact on the scale. Further external validation will be done in the near future in our next study.

There were also some other limitations to our study. Firstly, our sample included patients with SE, NCSE, and NORSE, but NCSE has been reported to have a worse prognosis than overt GCSE, with a mortality rate of 65% compared with 27% in the case of the latter [19]. In addition, some of the patients who had been admitted to various wards or the ICU with NCSE may have been neglected because, on the one hand, they did not present overt clinical manifestations and, on the other, the EEG was not used because this is not routine procedure for every hospitalized patient. Regarding our use of AEDs with SE, third-generation AEDs such as lacosamide and perampanel were introduced into our hospital relatively late, which might have had an effect on the overall outcome. Finally, we were unable to validate the EMSE, created in 2015, because the collection of the data used in our study, carried out from 2009 to 2020, was incomplete for several patients.

Additional prospective studies are needed to investigate whether the use of specific AEDs and the duration of a patient’s unresponsiveness contribute to a higher mortality rate, and whether these potential factors could be included in a predictive scale, which could be important to the clinical management of SE.

## 5. Conclusions

The clinical scales currently in use (the STESS, mSTESS, EMSE, and END-IT) helped us to predict the prognosis of SE. Our nSTESS, with the addition of the use of three or more AEDs within the first week, and the use of thiobarbiturates as clinical variables, could have a positive impact on the prediction of mortality rates.

## Figures and Tables

**Table 1:** Clinical characteristics of the survivors and non-survivors hospitalized in NCKU Hospital from 2009 to 2020.

Characteristics	Survivors (*N* = 53)	Non-Survivors (*N* = 6)	All	*p*-Value
Age–year (mean ± SD)	55.04 ± 21.33	56.67 ± 18.98	55.20 ± 20.95	0.859
Male sex–no. (%)	32 (60.4%)	4 (66.7%)	36 (61%)	0.769
Underlying diseases				
Meningioencephalitis–no. (%)	8 (15.09%)	0 (0%)	8 (13.6%)	0.583
Intracranial hemorrhage–no. (%)	15 (28.3%)	3 (50%)	18 (30.51%)	0.357
Seizure types				0.115
Focal impaired awareness	29 (54.72%)	4 (66.67%)		
Focal to generalized	4 (7.55%)	2 (33.33%)		
Generalized	18 (33.96%)	0 (0%)		
Nonconvulsive status epilepticus	2 (3.77%)	0 (0%)		
Categories of AEDs				
Valproic acid–no. (%)	31 (58.49%)	3 (50%)	34 (58.62%)	0.696
Levetiracetam–no. (%)	38 (71.7%)	6 (100%)	44 (75.86%)	0.157
Phenytoin–no. (%)	22 (41.51%)	3 (50%)	25 (43.1%)	0.696
Topiramate–no. (%)	11 (20.75%)	3 (50%)	14 (24.14%)	0.114
Lacosamide–no. (%)	1 (1.89%)	1 (16.67%)	2 (3.45%)	0.418
Perampanel–no. (%)	5 (9.43%)	2 (33.33%)	7 (12.07%)	0.313
Numbers of AEDs used in 1st week				0.016
1	15 (28.3%)	0 (0%)	15 (25.86%)	
2	25 (47.17%)	2 (33.34%)	27 (46.55%)	
3	10 (18.87%)	2 (33.34%)	12 (20.69%)	
4	2 (3.77%)	2 (33.34%)	4 (6.9%)	
5	1 (1.89%)	0 (0%)	1 (1.72%)	
Continuous infusion of sedatives				
Midazolam–no. (%)	31 (58.49%)	4 (66.67%)	35 (60.34%)	0.530
Propofol–no. (%)	4 (7.55%)	2 (33.34%)	6 (10.34%)	0.108
Thiobarbiturate–no. (%)	0 (0%)	2 (33.34%)	2 (3.45%)	0.009
STESS				0.117
0	1 (1.89%)	0 (0%)	1 (1.72%)	
1	10 (18.87%)	0 (0%)	10 (17.24%)	
2	16 (30.19%)	2 (33.34%)	18 (31.03%)	
3	19 (35.85%)	1 (16.67%)	20 (34.48%)	
4	5 (9.43%)	3 (50%)	8 (13.79%)	
5	2 (3.77%)	0 (0%)	2 (3.45%)	

AED, antiepileptic drug; SD, standard deviation; STESS, Status Epilepticus Severity Score.

**Table 2:** Components of Newly Modified STESS (nSTESS).

Clinical Feature	Score
**Consciousness**	
Alert or somnolent/confused	0
Stuporous or comatose	1
**Worst seizure type**	
Simple-partial, complex-partial, absence, myoclonic	0
Generalized-convulsive	1
Nonconvulsive status epilepticus in coma	2
**Age**	
<65 years old	0
≥65 years old	2
**History of previous seizures**	
Yes	0
Not or unknown	1
**Use of thiobarbiturate**	
Yes	1
No	0
**Numbers of used AEDs within 1st week**	
≤2	0
3	1
≥4	2
Total	0–9

**Table 3:** Capacity of the new scale (nSTESS) versus the STESS, mSTESS, and END-IT to predict mortality.

Scale	Sensitivity (%)	Specificity (%)	PPV (%)	NPV (%)	Overall Accuracy (%)	OR (CI 95%)	*p*-Value
STESS≥3	66.70%	50.90%	13.30%	93.10%	69.20%	2.077 (0.35–12.325)	0.352
mSTESS≥4	50.00%	56.60%	11.50%	90.90%	56.60%	1.304 (0.241–7.069)	0.543
END-IT≥3	50.00%	66.00%	14.30%	92.10%	57.70%	1.944 (0.356–10.625)	0.361
nSTESS≥4	83.30%	77.40%	29.40%	97.60%	86.80%	17.083 (1.816–160.683)	0.006

CI, confidence interval; nSTESS, newly modified Status Epilepticus Severity Score; mSTESS, modified Status Epilepticus Severity Score; NPV, negative predictive value; OR, odds ratio; PPV, positive predictive value; STESS, Status Epilepticus Severity Score; END-IT, Encephalitis-NCSE-Diazepam Resistance-Image Abnormalities-Tracheal Intubation.

## Data Availability

Data are available upon request from correspondence author, Chin-Wei Huang, Department of Neurology, National Cheng Kung University Hospital, College of Medicine, National Cheng Kung University
